# Grounding the right to live in the community (CRPD Article 19) in the capabilities approach to social justice

**DOI:** 10.1016/j.ijlp.2020.101551

**Published:** 2020

**Authors:** Emma Wynne Bannister, Sridhar Venkatapuram

**Affiliations:** a Department of Global Health and Social Medicine, King's College London, United Kingdom; b King's Global Health Institute, King's College London, Franklin-Wilkins Building, Stamford Street, London SE1 9NH, United Kingdom

**Keywords:** Capabilities approach, CRPD (Convention of the Rights of Persons with Disabilities), Human rights, Disability, Community

## Abstract

For advocates of the rights of persons with disabilities, particularly persons with mental disabilities, the human right to live in the community as an equal member is seen to be central and, often, even as the basis for all other human rights. Yet, despite its articulation in human rights law in the Convention on the Rights of People with Disabilities (CRPD), foundational issues about the right remain undertheorized and unclear. This paper brings to bear the capabilities approach, a normative framework about human well-being, social development and social justice, to this central concern in disability rights, mental health ethics, and international human rights law: protecting and respecting a person's right to live in a community as an equal. We argue that this human and moral right is best conceptualized as a capability to live in the community as an equal member. The capabilities approach provides this capability with a strong ethical framework and conceptual resources to guide reasoning and its practical realization.

## 1 Introduction

The following discussion makes a philosophical argument for the ‘capability to live in the community as an equal member’ (CLCE) as a moral foundation for the legal human right to live independently and be included in the community (Article 19 of the CRPD). With the capabilities approach (CA), a normative framework about human well-being, social development and social justice as the philosophical foundation, we articulate the CLCE. We bring to bear the CA to one of the central concerns in disability rights, mental health, ethics, and international human rights law— protecting and realizing a person's right and ability to live in a community as an equal member.

For advocates of the rights of persons with disabilities, including those persons with mental disabilities, the human right to live in the community (HRLC) is seen to be central and even the foundation to other rights and claims (Gradwohl, 2017; HRC, 2014). It is clearly articulated in Article 19 of the CRPD as the right to live independently and be included in the community (UNCRPD, 2006). The codification of the HRLC in international law is a signal achievement and reflects an important interest of persons with disabilities within legal debates. Yet, despite the articulation in human rights law, foundational issues about the right remain undertheorized and unclear. It is not made fully explicit what is so important about living in the community that it is seen to be a human right and one which should be enshrined in international law. Neither it is clear where this right comes from prior to it being articulated in the law.

There is an urgent need to clarify the ethical framework and foundations that can justify such a right as well as provide guidance on how individuals, institutions, and societies should act in relation to it. Without such a foundation or framework, there is a danger that the HRLC will be vulnerable to the long-standing and new criticisms of human rights (Hopgood, 2013; Moyn, 2018; Posner, 2014). Or that it will be caught up in legal debates and litigations about its scope, meaning and implications. As Tasioulas (2017) states about human rights more generally, “we cannot sustain our commitment to human rights on the cheap, by invoking only the law (…) Only a deeper justification can explain why we are right to embody them in the law…” (online). In addition to pointing to the law (e.g. CRPD), a philosophical and moral argument for the HRLC, including a coherent conception and justification, is something we need in order to be able to explain it to each other, wherever we are in the world. An argument to which no reasonable person could object offers a deep justification of the rights enshrined in human right law (Scanlon, 1998).

Grounding the HRLC in the CA is optimal as this approach is inclusive of all human beings (Nussbaum, 2006). In fact, the CA was developed in response to the persistent deprivations experienced by disadvantaged people and the weaknesses of the standard approaches to theorizing about social justice (Nussbaum, 2006; Sen, 1979). The CA also provides a substantive ethical foundation for human rights more generally. As Vizard, Fukuda-Parr, and Elson (2011) state, it emphasizes “the importance of viewing human rights as having legitimacy and validity within the ethical domain (rather than as simply being the ‘products’ of legal and institutional arrangements)” (p.2).

The claim for living in the community as an equal member is articulated here as the CLCE and grounded in the two ethical values of freedom and dignity, both foundational values of the CA. Whether grounded in either value, it is a basic or central human capability (CHC) and part of a minimally decent or flourishing human life. It is morally valuable as a constituent part of wellbeing because of human beings' inherent sociability, and instrumentally valuable as it helps pursue other capabilities that make up a decent and flourishing life. Such a right (and capability) is important for all humans and is relevant and crucial for a diverse range of disadvantaged people.

However, it is particularly salient for persons with mental disabilities because they have often been excluded, either physically through institutionalization or socially through not being treated as equal members. Among the many types of disability, mental impairments have historically been among the most neglected conditions in health and social policy (Black, Laxminarayan, Temmerman, & Walker, 2016). This has rendered those who suffer from them disabled, with poor quality of life, vulnerable and often dependent on others (Lang, Kett, Groce, & Trani, 2011). Moreover, the ‘moral status’ of persons with mental disabilities who have cognitive impairments has long been insecure in Western philosophical discussions (Putnam, Wasserman, Blustein, & Asch, 2013; Wasserman, Asch, Blustein, & Putnam, 2012; Wilkinson, 2008). The belief that people with mental impairments are less than morally equal or not fully human makes them more vulnerable to being excluded and deprived of living as equal members of human communities.

The following discussion is organized into four sections. Section 2 introduces the capabilities approach while focusing on its relevance to the present argument. Section 3 discusses the CRPD and Article 19. Section 4 brings together the CA and Article 19 of the CRPD arguing how the CA can provide the ethical basis for a human right to the capability to live in the community as an equal member. It also presents some possible misinterpretations and critiques of the CLCE argument. Finally, in section 5, a conclusion to the discussion is presented.

In line with the social model of disabilities and the CA, which will be further discussed in the paper, the term ‘disabilities’ will be used throughout the text to refer to those conditions where persons with impairments are disabled by the social, economic and environmental conditions (external conditions in capabilities terminology) to which they are exposed. The terms mental impairments and illnesses refer to the individual biological or psychological conditions (internal conditions in capabilities terminology) that affect a person's mental health at any given time in their life (Burchardt, 2004; Oliver, 1983). In this paper, following recommendations made in the CRPD, ‘persons with disabilities’ will be the preferred term when speaking of individuals with impairments who become disabled when interacting with several social barriers (UNCRPD, 2006). This terminology is used in section 3 as the CRPD focuses on disabilities more generally. However, in this paper, the focus is on persons with mental disabilities. That is, those individuals with mental impairments who become disabled from interacting with external conditions. Although the proposed CLCE is relevant to all humans, we consider it is particularly salient to persons with mental disabilities who have often had said capability denied primarily through institutionalization and unequal treatment.

## 2 Capabilities approach

The CA is an analytical and ethical framework that redefines the concepts of human well-being, equality and freedom as well as shifts the paradigm of social development and progress. Some of the foundational concepts of the CA can be traced back to Aristotle's understanding of human flourishing, Marx's conceptualization of functionings, and Adam Smith's idea of relative poverty (Sen, 1983, Sen, 1999c; Wells, 2012). The initial presentation of the theory of human capabilities was in the 1979 Tanner Lecture on Human Values entitled “Equality of what?” given by the economist Amartya Sen, who was later awarded a Nobel Prize for his contributions to welfare economics. In the lecture, he identified the conceptual shortcomings of measuring inequality, poverty and well-being in the then prevailing terms of income or material resources, negative liberties, basic needs, or utility (happiness). As an alternative, he put forward the concepts of human “capabilities”, what individuals are able to be and do in their daily lives, and capabilities equality/equity (Sen, 1979).

The CA motivates describing and evaluating a person's well-being or quality of life in terms of her practically possible opportunities (‘capabilities’) to achieve various outcomes – “beings and doings” –(‘functionings’) that make up a good or flourishing life. From a CA perspective, social development or progress —in low, middle, and high-income countries—is the expansion of such real opportunities of people. The CA places individuals, their values, and their freedom of choice of pursuing opportunities to be and do certain things at the center of analysis. Importantly, the CA envisions well-being as something that is made up of different kinds of beings and doings, which are each valuable and not to be traded off (Nussbaum, 2002b, Nussbaum, 2003, Nussbaum, 2006, Nussbaum, 2011; Sen, 1979, Sen, 1992, Sen, 1999a, Sen, 1999b, Sen, 1999c).

From the CA perspective, capabilities are not just internal characteristics of a person and they are not individual capacities; capabilities are formed through the combination of internal and external conditions. The internal factors encompass individual, often biological characteristics. These can include factors such as impairments, illness, gender or age. All of which result in diverse biological needs. Whereas external conditions encompass both the physical and social environments. These cover environmental diversities such as climatic circumstances; institutional variations such as the different public services available in different contexts; varied relational perspectives which relate to commodity requirements established by social norms, conventions and customs; and distributional factors, such as how goods are distributed among groups including families (Deneulin & Shahani, 2009; Sen, 1999c). As a result, the CA “provides a way of conceptualizing the disadvantage experienced by individuals in society, which emphasizes the social, economic and environmental barriers to equality” (Burchardt, 2004, p.735).

Martha Nussbaum, who collaborated with Sen for some years has also made significant contributions to the CA. She has developed a theory of ten CHCs and grounds them in human dignity. This contrasts to Sen's grounding of the CA in the value of freedom. Nussbaum understands human beings as ethical subjects who, in line with Aristotelian and Marxian thought, are beings that are needy, sociable and have the ability to reason (Formosa & Mackenzie, 2014; Nussbaum, 2002b). These characteristics are the basis of her conception of dignity and are what “gives rise to moral claims on others for the protection and development of some basic capabilities” (Venkatapuram, 2014 p.410). She argues that dignity's relation to other notions which include respect, agency and equality is what makes it important to human lives (Nussbaum, 2011). Her theory of basic justice, or pre-political (read pre-legal) entitlements, consists of central capabilities that all people should possess, and up to a certain threshold (Nussbaum, 2011).

Nussbaum's ten central capabilities make up a life with human dignity. Her list includes capabilities for 1) life, 2) bodily health, 3) bodily integrity, 4) senses, imagination and thought, 5) emotions, 6) practical reason, 7) affiliation, 8) other species, 9) play, and 10) control over one's environment (Nussbaum, 1997, Nussbaum, 2011). The list of central capabilities can be added to and adapted to fit different contexts. However, societies cannot neglect any of the basic ten, and the whole population must have or be brought up to a specified threshold of each of these central capabilities. Given the plurality of values and variation in social priorities that exist in different countries, the various thresholds can and should be established by each nation (Nussbaum, 2011). Nussbaum has, however, not explicitly stated how each threshold is to be determined beyond stating that it should be achieved through expertise and democratic debate. Given the ten capabilities constitute only a minimal human flourishing, Nussbaum's is a partial or basic theory of justice. It is not a full theory or conception of social justice, as it is “a social-minimum approach… it doesn't say what should be done about inequalities above its rather ample threshold” (Nussbaum, 2009, p.332).

Over the four decades since the lecture and following from the many publications by Sen on the subject, an extensive interdisciplinary school of thought has developed around the initial idea of human capabilities. Scholars, researchers, and practitioners have developed and used the approach as an analytical and normative framework in a broad range of fields. The analytical contribution of the CA is the measuring of wellbeing in terms of capabilities, which is argued to be more coherent than measuring resource holdings, liberties, basic needs, or utility. Examples of this application include the analysis and measurement of national wellbeing, poverty and inequality, the modelling and evaluation of development projects, and the assessment of living standards (Alkire, 2002, Alkire, 2005; Anderson, 1999; Arneson, 2002; Brighouse, & Robeyns, I. (Eds.)., 2010; Deneulin, 2003; Wolff, & de-Shalit, A., 2007). And its normative or ethical contribution is the philosophical justification for every individual's moral claims to capabilities, as well as for a conception of a good society as one that protects and expands human capabilities (Robeyns, 2005, Robeyns, 2011, Robeyns, 2017). This has resulted, for example, in the prescription and design of welfare policies.

### 2.1 Disabilities in the CA

In most articulations of the capabilities approach, health capability(ies) is considered one of the basic dimensions of a good life (Nussbaum, 2011; Sen, 1999c). Multiple scholars have used the CA to explore and analyze matters of health and its centrality to wellbeing (Burchardt, 2004; Coast, Smith, & Lorgelly, 2008; Entwistle, Cribb, & Owens, 2018; Ruger & Mitra, 2015; Venkatapuram, 2011). Among health topics, philosophical arguments from the CA have also been put forward considering disabilities in relation to distributive justice. Acknowledging the individual diversity of human beings and how they are each uniquely situated in daily life are central tenets of the CA. As a result, persons with disability are considered equal from the start of theorizing about equality and social justice. Both Sen and Nussbaum have included disability in many of their writings, including in the initial Tanner lecture (Nussbaum, 2006; Sen, 1979, Sen, 1999b, Sen, 2009).

Since the CA focuses on what people are capable of being and doing, and that one of the main tenets of the CA is that diversity is intrinsic to human beings, understanding how individuals convert resources (external conditions and commodities) into capabilities and functionings that are valuable to wellbeing is central (Robeyns, 2011). Within the CA the concept of ‘conversion factors’ reflects the varying abilities people have to turn their internal characteristics and external conditions into beings and doings they value. This is true for all individuals and is particularly salient for persons with disabilities. For example, a person with a severe mental impairment might require more resources to cover costs of full-time care which enables her to enjoy the same level of well-being as someone without a mental disability who does not require care. Therefore, the CA enables viewing disability or capabilities constraints as being produced through a combination of both internal and external factors that impede the person to function in society fully, rather than solely as the product of biology or individual duties (Burchardt, 2004; Harnacke, 2013; Mitra, 2006, Mitra, 2018).

Every human being will be constrained by their biology at various times throughout their lives. As such, the CA understands that disabilities might be temporary or permanent, and also claims that people should be entitled to basic capabilities regardless (Nussbaum, 2002a) How disabled, in-capable, or unfree people are to pursue a decent life is determined by the combination of their internal and external conditions and the temporality of these (Burchardt, 2004; Mitra, 2018; Nussbaum, 2002a; Ruger & Mitra, 2015). In light of this view, the CA has proven to be an important and productive framework to identify and measure how persons with disabilities, including mental disabilities, require different types and amounts of resources, based on their conversion factors, to achieve the same (read equal) capabilities and functionings as non-disabled persons (Burchardt, 2004; Sen, 1994). This understanding has also helped expose legal rights violations in the barriers people with disabilities face in everyday activities and in accessing support (Szmukler & Bach, 2015).

This evidences that the CA shares several commonalities with the social model of disability (Burchardt, 2004; Dubois & Trani, 2009; Mitra, 2006). Namely, disabilities are limitations on opportunities to be part of the community in equal standing to others. The social model maintains that disabilities are produced from physical, social and economic environmental factors to which people are subjected (Burchardt, 2004; Oliver, 1983). The distinction between disability, which involves the social, economic and environmental or external conditions, and impairment, which refers to the biological or internal condition, is the seminal contribution of the social model of disability (Oliver, 1983). It places the blame of non-participation in the community on the social conditions rather than on the individual who has an impairment (Burchardt, 2004). In comparison, the CA starts from the position that people in a society will each have different needs and abilities (Harnacke, 2013), these change over the life course, and some will have severe impairments throughout their life. Ignoring such diversity both in theorizing and practical policymaking is seen to be the reason for much preventable suffering as well as toleration of inequalities in wellbeing.

Sen, Nussbaum, and other CA advocates have clearly asserted that persons with disabilities should be considered on an equal standing within a given society (Nussbaum, 2006; Sen, 1999b, Sen, 2009). Disability rights advocates find the CA compelling because people with disabilities are not an afterthought to the CA's conceptualization of wellbeing equity and justice and are central to the way the framework was developed and functions. Also, the social disability model says that social conditions are what makes people disabled, but it does not give an ethical argument for why social conditions should enable people. The CA makes an argument for capabilities (abilities), and then says it is unjust for social conditions to neglect or constrain the capabilities of people. The CA extends positive obligations for respecting human rights to support and defend these, in addition to negative duties to leave individuals to pursue their diverse conceptions of life (Sen, 2009, Vizard, 2010). The CA is both analytically helpful and normatively powerful. As a matter of justice, society must refrain from harming/interfering individuals and has positive obligations to help expand capabilities.

## 3 CRPD & Article 19

Human rights law is an important framework that has been harnessed to address the interests of persons with disabilities. Although the 1948 Universal Declaration of Human Rights (UDHR) aimed for inclusivity and providing rights for all people, at that time persons with disabilities “were still considered as ‘objects’ of charity, medical treatment and social welfare” (UN., 2018). Most recently, the CRPD constitutes a paradigm shift in comparison to other human rights treaties. It acknowledges persons with disabilities, both mental and physical, as ‘subjects’ with rights, “who are capable of claiming those rights and making decisions for their lives based on their free and informed consent as well as being active members of society” (Quinn & Doyle, 2012; UNCRPD, 2006).

Indeed, there is a long history of persons with disabilities being incorporated into numerous international rights agreements prior to the CRPD. However, the perception of persons with disabilities as objects of social concern, rather than as individuals with agency has persisted. And this perception has led to severe deprivations and violations of the moral rights of persons with disabilities. Additionally, it was still unclear what specific or unique claims persons with disabilities have, and what the obligations the national governments and others have to ensure these claims or rights are realized (UN., 2007). Disability rights advocates and the international community sought to address the lack of inclusion, dehumanization and persistent discrimination of persons with disabilities through drafting a new human rights instrument (UN., 2007).

As such, the CRPD is “the latest addition to the body of core international human rights instruments” (UN., 2007, p.5). It comprehensively identifies that persons with disabilities are rights holders and full citizens who make valuable contributions to society. The purpose is “to promote, protect and ensure the full and equal enjoyment of all human rights and fundamental freedoms by all persons with disabilities, and to promote respect for their inherent dignity” (UNCRPD, 2006, online). Although the CRPD does not define disability, it states that persons with disabilities include persons with physical, mental, intellectual or sensory disabilities (UNCRPD, 2006).

The CRPD aims to establish that persons with disabilities are entitled to human rights by nature of being human and offers an unprecedented level of protection to these persons. The Convention does not put forth any new human rights and rather aims at clarifying the duties States have to respect and ensure the equal enjoyment of existing human rights by persons with disabilities. As such, it identifies where modifications need to be made and where protection (read support) needs to be put in place for persons with disabilities to be able to access the human rights that they have had continuously violated (UN., 2007). The Convention, as a binding international treaty and legal human rights instrument with a social development dimension, seeks to lift barriers that hinder persons with mental disabilities’ ability to fully participate ‘as equal members’ in societies worldwide (Szmukler, Daw, & Callard, 2014; UNCRPD, 2006).

In attempting to lift these barriers, beyond arguing for persons with disabilities as subjects, the CRPD also differs from previous human rights articulations in several ways. First, it establishes clear responsibilities and actions for states in order to respect, protect and ensure these rights. This is to be done through incorporating these rights into the national legal system, as well as through raising awareness, increasing service accessibility, providing protection in humanitarian crises, ensuring accessibility to justice, enabling mobility and rehabilitation, and monitorization of policies and statistics (HRC, 2014; UN., 2007, UN., 2018; UNCRPD, 2006). Second, it establishes the State has the responsibility to be inclusive of persons with disabilities in all development interventions, both on a national and international level. Third, the CRPD requires that States consult with organizations that represent persons with disabilities on matters that affect them, be it through policies aimed specifically with them in mind, or those that might affect them. And fourth, the CRPD also identifies the importance of international cooperation through shared knowledge, capacity building, and economic assistance (HRC, 2014; UN., 2007, UN., 2018; UNCRPD, 2006).

Importantly, consulting persons with disabilities reflects the right of persons with disabilities to control how they want to live as well as participate in the community (CRPD Committee, 2017). In line with this claim, the CRPD argues that “independent living and an inclusive life in the community are ideas that are inextricably linked with the disability rights movement” (CRPD Committee, 2017, p.1). Therefore, the CRPD is also innovative by explicitly including Article 19, the right to live independently and in the community. In fact, this right has been described as being the essence of the entire convention (Gradwohl, 2017; HRC, 2014; Quinn & Doyle, 2012; Simplican, Leader, Kosciulek, & Leahy, 2015) and “a substantive means for the realization of other rights” (HRC, 2014). Furthermore, the converse is also asserted; the Convention's core principles enunciated in Article 3, “(…) particularly respect for the individual's inherent dignity, autonomy and independence (Art. [a]), and the full and effective participation and inclusion in society (Art. 3[c])” are the foundation of Article 19 (CRPD Committee, 2017).

Social exclusion, and often being physically isolated from family and community, did not seem to be a prominent concern for non-disabled individuals. Therefore, a right to protect against this was not explicitly expressed in human rights law. Living in the community looks to have been a starting assumption in previous human rights declarations and treaties. The CRPD transforms that assumption into an explicit and central right by emphasizing that “living as a part of our communities – from local to global – serves as the basis for everything we do in life” (CECHR, 2012). Furthermore, it is based on the idea that human flourishing is more likely when we are able to develop in a community with others (Quinn & Doyle, 2012). Article 19 thus “embodies a positive philosophy, which is about enabling people to live their lives to their fullest, within society” (CECHR, 2012).

This right is a result of the CRPD's aim to ensure rights already available to persons without disabilities to persons with disabilities no matter how severe the disability is or how intensive the support needs are (Hammarberg, 2012). This right to live independently and be included in the community is articulated in Article 19 of the CRPD as follows:

“States Parties to the present Convention recognize the equal right of all persons with disabilities to live in the community, with choices equal to others, and shall take effective and appropriate measures to facilitate full enjoyment by persons with disabilities of this right and their full inclusion and participation in the community, including ensuring that: (a) Persons with disabilities have the opportunity to choose their place of residence and where and with whom they live on an equal basis with others and are not obliged to live in a particular living arrangement; (b) Persons with disabilities have access to a range of in-home, residential and other community support services, including personal assistance necessary to support living and inclusion in the community, and to prevent isolation or segregation from the community; (c) Community services and facilities for the general population are available on an equal basis to persons with disabilities and are responsive to their needs” (UNCRPD, 2006, online).

The different clauses of Article 19 identified aim at protecting different aspects of this right. Clause (a) is focused on choice and the conception of persons with disabilities as autonomous individuals with the capacity of making their own choices (CECHR, 2012). It also emphasizes the importance of not being restricted to a specific living arrangement. Such a freedom is aimed at avoiding forced or unnecessary institutionalization (Quinn & Doyle, 2012). Clause (b) expresses the importance of individualized community support. It reflects a conception of independent living, where independence is not meant living in a self-sufficient manner at a distance from others. Instead, it argues for support systems, which the individual can choose and be in control of, to be put in place (CECHR, 2012). Finally, clause (c) focuses on equality for persons with disabilities when it comes to public services that exist in the community. It calls for increased social connectedness and for existing social services to be inclusive of persons with disabilities; disabled and non-disabled citizens should be able to access public services as equal members of society and citizens (Quinn & Doyle, 2012).

These clauses highlight the areas in which the State needs to take action to protect the right to live independently and be included in the community. Nevertheless, no easy solutions follow on how to implement this right. It has been argued that the CRPD is an ambitious treaty and that despite the ratification of the CRPD by states, it is difficult to hold these accountable of implementation (De Búrca, 2010; Molas, 2016). It is often the case that adhering to the CRPD will require States to overhaul their infrastructure and legislation, making implementation costly and complicated, particularly in some contexts. This seems especially so concerning the rights of persons with mental disabilities (McSherry, 2014).

Further, in some situations, there is a lack of understanding of disabilities from a human rights perspective despite the political discourse stating the contrary (Lang et al., 2011). Among the main challenges identified in implementing the CRPD, Lang et al. (2011) argue that rights claims continue to be seen as within the realm of charity due to countries having “(…) limited knowledge, understanding, and in some case [limited] support for the fundamental principles and practices underpinning human rights” (p.215). Consequently, human rights violations of persons with physical and mental impairments continue to persist and are reported around the world (Szmukler & Bach, 2015).

Given these issues, a conceptual and ethical framework for Article 19 of the CRPD, including a strong justification beyond or prior to international human rights law, is necessary to ensure that persons with mental impairments can turn this foundational right into something meaningful in their daily lives. The CLCE grounded in the capabilities approach has the potential to help individuals and communities understand and realize this right, as well as institutions and governments recognize its place in policymaking. The justification for this right and where it comes from prior to legal discourse is useful for governments to understand why it is important. Thus, the CA can give meaning to the legal human rights articulated in international treaties. Similarly, the concept of a threshold in the capabilities approach can allow governments to work toward enabling a minimal level of the CLCE that is in line with a dignified life given their resource constraints and contextual priorities.

The argument can also assist disability advocates to access the ethical language and resources of theories of justice, including debates about rights, human rights, equality, and wellbeing. The conceptual clarity on what is meant by disability offered by the CA, as well as the social model of disability, enables advocates to explain to governments the contents of human rights documents. This ethical language can also help to explain the values that ground the CLCE. Freedom and dignity are values that can be more widely supported in different contexts than international legal documents that result from political deliberation.

## 4 Ethical foundations of the human right to live in the community (HRLC)

The CA can be a useful framework from which to address issues raised by the CRPD. In fact, there is some evidence that the CRPD was informed by the CA, just as it has also influenced many other recent international treaties. The notion of human flourishing, central to the CA, has also been claimed to be “deeply embedded in the logic of the UN CRPD” (Quinn & Doyle, 2012). But much of the conceptual clarity of the CA may have been lost in the final texts of the convention which are often achieved through difficult political and legal negotiations typical of international proceedings (Basser, Jones, & Rioux, 2011; Venkatapuram, 2014). The following section aims to present the CA's conceptualization of the right to live independently and in the community as an equal member, and its centrality to human well-being.

Both Sen and Nussbaum have, in some way, discussed the centrality of the capability to live in the community to the CA, even though not explicitly articulating it as a basic capability. Furthermore, Sen's focus on freedoms and Nussbaum's focus on dignity both provide good starting points for the ethical underpinnings of Article 19. All reasonable human beings value freedoms to design, pursue and revise their life plans (Sen, 1992; Venkatapuram, 2013). The CA articulates clearly the importance of having real opportunities to be and do what one values, incorporating both the importance of autonomy and the understanding that providing social conditions that create capabilities, specific to the diversity of the individual, enables autonomy and is the obligation of the surrounding society, including the State apparatus.

While Sen has not identified a universal and core set of human capabilities, his discussion on the freedom to live in the community as an equal is evidence of its importance (Sen, 2002). He considers individuals to be inherently social and stresses that individual freedoms depend on social arrangements (Deneulin & Ritchie, 2006; Sen, 2002). It is clear from Sen's writings on capabilities and wellbeing that he considers social integration as a capability that is widely valued (Nussbaum & Sen, 1993). That is, sociability is an important freedom, and social interactions create certain freedoms. Furthermore, Sen argues that social exclusion can lead to other forms of deprivation. For example, being excluded from social relations can lead one to not have the opportunity to be employed and this may lead to impoverishment that further constrains one's opportunities (Sen, 2000).

Sen has also made several arguments about shame and being able to participate in society. Sen speaks about the existence of certain moral rights, that might not be articulated in law, which a person requires to feel included in the community. He illustrates this with the example of the importance of protecting a person's freedom of speech by ensuring that persons with stammers are not ridiculed (Sen, 2009). He also makes the point that often, being a member of a community requires having similar capabilities as all the others in the community in order to avoid shame and enable social interactions (Sen, 1983). All of these above ways that community is important to human wellbeing, Sen argues, are related to “the Aristotelian understanding that the individual lives an inescapably ‘social’ life” (Sen, 2000, p.4).

However, despite highlighting the importance of being included in the community as an equal member, Sen claims that the capabilities that are identified as valuable should be arrived at through public deliberation. Further, he makes a practical argument about the link between capabilities and human rights. He argues that capabilities are basic freedoms that come to be considered human rights when they achieve a threshold of relevance, −which includes both the importance of this freedom and the possibility of realizing it. Such a relevance must also be arrived at through public reasoning, where the society's value of a particular kind of freedom will justify its incorporation into human rights (Sen, 2009).

On the other hand, Nussbaum asserts her CHCs to be pre-political entitlements of human rights. She understands these basic capabilities as universal moral claims that can provide the grounding of legal claims enshrined in state constitutions and international human rights treaties. She argues, however, that a right when it is ‘put down on paper’ is not enough. Only when effective measures are in place for people to achieve them do these become real (Nussbaum, 2002b).

Regarding being included in the community, Nussbaum has argued that “human beings enjoy status dignity by virtue of their humanity; that is, by virtue of their membership of, first, the human species and, second, the human community” (Formosa & Mackenzie, 2014). And, in her list, she has explicitly included the capability for affiliation (Nussbaum, 1997, Nussbaum, 2011). Moreover, she expresses the view that although all capabilities on her list are of central importance, affiliation, as well as practical reason, are “of special importance because they both organize and suffuse all the other capabilities, making their pursuit truly human” (Nussbaum, 1995, Nussbaum, 1997). The capability of affiliation, according to Nussbaum, aims to give people the freedom of:

“(A) being able to live with and toward others, to recognize and show concern for other human beings, to engage in various forms of social interaction; to be able to imagine the situation of another. (Protecting this capability means protecting institutions that constitute and nourish such forms of affiliation, and also protecting the freedom of assembly and political speech.) (B) Having the social bases of self-respect and nonhumiliation; being able to be treated as a dignified being whose worth is equal to that of others. This entails provisions of nondiscrimination on the basis of race, sex, sexual orientation, ethnicity, caste, religion, national origin” (Nussbaum, 2011, p.34).

Although there is some disagreement among CA scholars about establishing a list of CHC, it is clear from the above, that the CLCE is identified in both Sen and Nussbaum's approaches. Building on this work, the CLCE can be conceptualized as follows. Grounded in freedom or dignity, the capability to live in the community as an equal member is an important aspect of a flourishing life. Such a capability is as important, if not crucial, to the wellbeing of individuals with disabilities as it is for non-disabled individuals. And, the capability remains important whether the disability is permanent or temporary.

It consists in the freedom to decide on one's living arrangements within the community, as well as the freedom to participate as an equal member in society in line with a life worth dignity. That is, the CLCE entails the freedom to be included in the community as an equal with adequate support from the state and other members of the community. Support must be provided considering both internal and external factors that allow for a person to achieve the CLCE. As such, it represents both social and physical inclusion within the community and requires support from the social and physical environment, as well as resources. It produces duties for social support, involving an adequate level of care and the availability of caretakers to support persons with disabilities and enable this freedom. Similarly, it requires support from the physical environment through institutions and infrastructure being inclusive and responsive to the needs of persons with disabilities, including persons with mental disabilities. Additionally, given the diverse abilities humans have to convert resources into beings and doings, there is a duty to provide adequate resource distribution that enables persons with mental disabilities to achieve the same level of social and physical inclusion within the community as that of persons who do not have a disability.

What is important is that the CLCE is a component of wellbeing, which is both intrinsically and instrumentally valuable. It is intrinsically valuable because humans are inherently social and needy beings (Nussbaum, 2002b; Sen, 2002). The freedom to be socially and physically included in the community is part of what makes up a life of equal human dignity. On the other hand, the CLCE is instrumentally valuable because this capability enables other basic human capabilities to be realized. Living in the community as an equal member allows persons with disabilities to participate in social arrangements and interactions on which other valuable freedoms depend.

One source of misinterpretation has been through the translations of the HRLC into other languages. Mladenov (2013), found that independent living had been translated as ‘self-standing’ and ‘autonomous’ in several languages. This might result in policies that advocate for ‘deinstitutionalization’ without providing support in the community to exercise everyday activities while living independently. Indeed, independent living can be interpreted in a variety of ways in English as well. A clear capability to live in the community as an equal member produces duties to support a person with disabilities to have the freedom to live a life worth dignity.

The CLCE argument is not meant to achieve deinstitutionalization with the result of persons being left to fend for themselves. Such a view “rest on a mistaken notion that living in the community is solely about physical placement in the community” (CECHR, 2012). Moreover, these mistaken interpretations do not take into account that “countless more people with disabilities are physically located in their communities, but barred from meaningful participation (…) because either services are not available or communities are organized in ways that exclude them from participation” (CECHR, 2012).

Thus, true participation includes relying on social solidarity, as being included in the community goes hand in hand with belonging to families and other groups that can provide support (Deneulin & Ritchie, 2006; Stewart, 2005; Trani & Dubois, 2011). The CLCE reflects a full inclusion of a person with a disability in their environment as it makes the argument for persons to live as equal members, in other words, in equal standing with others. That is, they must not be expected to be fully autonomous and must not be isolated from participating in the community as this would not be expected of a person who does not have a disability. This capability embodies the understanding that for persons with disabilities to live a decent or minimally flourishing life they must have equal freedoms as others.

Some of the criticisms the argument for the capability to live in community as an equal member could receive include the point that scarcity of resources makes it incoherent to assert something as a moral right to a basic capability when it cannot immediately be realized. Or that the capabilities framework is an imposition of Western values and reasoning.

The resource scarcity critique is also made of human rights approaches. That is, something cannot be a right or a human right if it cannot be immediately be realized, Framing this right as a capability allows for making the claim that persons are morally entitled to live in the community as equal members, and for arguing for the corresponding duty nation-states have to take responsibility in ensuring that it is realized. Furthermore, Nussbaum's conceptualization of a threshold affects the resource critique in realizing the capability to live in the community as an equal member. Nussbaum's approach allows for nations to establish the threshold of each capability, reflecting minimal human dignity, ensuring that all persons have access to this capability. Where a country does not have the resources to help their citizens to reach the threshold, international assistance is required. This will entail nation-states with more resources to support those who cannot provide basic threshold capabilities for their citizens as a matter of global justice (Nussbaum, 2009).

While some scholars state that the CA, like human rights, reflects Western values (Dean, 2009; Stewart, 2001), Nussbaum has claimed that the CA is able to avoid this critique. Nussbaum's approach is based on an account of human flourishing which is historically grounded and based on an empirical inquiry of what persons in different societies value, and, primarily, the result of philosophical theorizing (Nussbaum, 1992, Nussbaum, 2011). Her list of 10 CHC is one that has evolved over years of cross-cultural discussions, which she argues can lead to overlapping consensus on the items included (Nussbaum, 2000). Furthermore, she argues that given it is a social-minimum approach, her list enables multiple ways of life, which can be supported in all societies, including pluralistic societies. The capabilities on her list may be constructed and realized differently in different societies rather than imposing a particular conception of the good life in all contexts (Nussbaum, 1992, Nussbaum, 2000; Nussbaum, 2006; Uyan-Semerci, 2007; Vasbist, 2010). Indeed, as Uyan-Semerci (2007) has noted, “the approach derives its strength because it genuinely attempts to develop an understanding of human diversity” (p.203). Moreover, Sen claims that freedom, the basis of the CA, has been found to be a value that is universally shared across different cultures and societies (Sen, 1999c). Similarly, Robeyns (2017) has stated that since the CA terminology is not tied to a western or colonial power it is not considered an instrument of domination as has been claimed with human rights.

## 5 Conclusion

Disability rights advocates have pressed for the human right to live independently and in the community for persons with disabilities, as they claim this right is foundational to other rights. Indeed, this right (and capability) is important for all humans and particularly pressing for vulnerable groups, including persons with disabilities. Despite a longstanding history of persons with disabilities being the focus of various international rights agreements, the CRPD represents a paradigm shift and achievement for disability rights movements. However, scholars and practitioners have identified “a need for further ethical argumentation in justifying why and how such a paradigm shift exists” (Celik, 2017, p.935). Questions such as what is important about living in the community and where this right comes from prior to the law remained unclear.

In answering that call, we have sought to ground the right to live independently in the community in the capability approach as it can serve as the ethical framework and foundations that can justify such a right. This right was conceptualized as a capability and it was shown it is grounded in the two values of freedom and dignity. The capability to live in the community as an equal member consists in being able to decide on one's living arrangements within the community, as well as having the freedom to participate as an equal member in society in line with a life worth dignity.

As such, the CLCE entails the freedom to be included in the community as an equal with adequate support from the state and other members of the community to do so. In specific, it was made clear that this capability is not simply an argument for deinstitutionalization of persons with mental impairments without proper support. Rather it is a positive argument for supporting persons with mental disabilities to develop the CLCE which will enable real inclusion within the community.

## Funding

Emma Wynne Bannister is supported by The National Council for Science and Technology of Mexico (CONACYT) and The Sir Richard Trainor Scholarship. Funding bodies were not involved in the preparation of this article. Funding was received from the Wellcome Trust funded Collaborative Award, “Mental Health and Justice” (203376/Z/16/Z).

## References

[bb0005] Alkire S. (2002). Valuing Freedoms.

[bb0010] Alkire S. (2005). Why the capability approach?. Journal of Human Development.

[bb0015] Anderson E. (1999). What is the point of equality?. Ethics.

[bb0020] Arneson R. (2002). Egalitarianism.

[bb0025] Basser L.A., Jones M., Rioux M.H. (2011). Critical perspectives on human rights and disability law. *Brill*.

[bb0030] Black R.E., Laxminarayan R., Temmerman M., Walker N., Patel V., Chisholm D., Dua T., Laxminarayan R., Medina-Mora M.L. (2016). Disease control priorities. *Mental, neurological, and substance use disorders*.

[bb0035] Brighouse H., Robeyns, I. (Eds.). (2010). Measuring justice: Primary goods and capabilities.

[bb0040] Burchardt T. (2004). Capabilities and disability: The capabilities framework and the social model of disability. *Disability & Society*.

[bb0045] CECHR (2012). The right of people with disabilities to live independently and be included in the community.

[bb0050] Celik E. (2017). The role of CRPD in rethinking the subject of human rights. The International Journal of Human Rights.

[bb0055] Coast J., Smith R., Lorgelly P. (2008). Should the capability approach be applied in health economics?. *Health Economics*.

[bb0060] CRPD Committee (2017). Draft General Comment on the right to living independently and be included in the community.

[bb0065] De Búrca G. (2010). The EU in the negotiation of the UN disability convention. *European Law Review*.

[bb0070] Dean H. (2009). Critiquing capabilities: The distractions of a beguiling concept. Critical Social Policy.

[bb0075] Deneulin, Ritchie A. (2006). Affiliation and community agency: The case of broad-based community organising in London. Presented at the 5th International Conference on the Capability Approach: Knowledge and Public Action at UNESCO.

[bb0080] Deneulin S. (2003). Examining Sen’s capability approach to development as guiding theory for development policy (doctoral dissertation).

[bb0085] Deneulin S., Shahani L. (2009). Introduction to the human development and capability approach: Freedom and agency.

[bb0090] Dubois J.-L., Trani J.-F. (2009). Extending the capability paradigm to address the complexity of disability. ALTER - European Journal of Disability Research / Revue Européenne de Recherche Sur Le Handicap.

[bb0095] Entwistle V.A., Cribb A., Owens J. (2018). Why health and social care support for people with long-term conditions should be oriented towards enabling them to live well. *Health Care Analysis: HCA: Journal of Health Philosophy and Policy*.

[bb0100] Formosa P., Mackenzie C. (2014). Nussbaum, Kant, and the capabilities approach to dignity. *Ethical Theory and Moral Practice*.

[bb0105] Gradwohl C. (2017). The right to live in the community as the right to have rights.

[bb0110] Hammarberg T. (2012). Annual activity report 2011 Commission for Human Rights of the Council of Europe.

[bb0115] Harnacke C. (2013). Disability and capability: Exploring the usefulness of Martha Nussbaum’s capabilities approach for the UN disability rights convention. *The Journal of Law, Medicine & Ethics: A Journal of the American Society of Law, Medicine & Ethics*.

[bb0120] Hopgood S. (2013). The endtimes of human rights.

[bb0125] HRC (2014). Thematic study on the right of persons with disabilities to live independently and be included in the community (No. Twenty eighth session).

[bb0130] Lang R., Kett M., Groce N., Trani J.-F. (2011). Implementing the United Nations convention on the rights of persons with disabilities: Principles, implications, practice and limitations. *ALTER - European Journal of Disability Research / Revue Européenne de Recherche Sur Le Handicap*.

[bb0135] McSherry B. (2014). Mental health laws: Where to from here?. *Monash University Law Review*.

[bb0140] Mitra S. (2006). The capability approach and disability. Journal of Disability Policy Studies.

[bb0145] Mitra S. (2018). Disability, health and human development. New York: Palgrave Macmillan US..

[bb0150] Mladenov T. (2013). The UN convention on the rights of persons with disabilities and its interpretation. *ALTER - European Journal of Disability Research / Revue Européenne de Recherche Sur Le Handicap*.

[bb0155] Molas A. (2016). Defending the CRPD: Dignity, flourishing, and the universal right to mental health. The International Journal of Human Rights.

[bb0160] Moyn S. (2018). Not enough: Human rights in an unequal world.

[bb0165] Nussbaum M. (1992). Human functioning and social justice: In defense of aristotelian essentialism. *Political Theory*.

[bb0170] Nussbaum M., Altham J.E.J, Harrison R. (1995). Aristotle on human nature and the foundations of ethics. *World, mind, and ethics: Essays on the ethical philosophy of Bernard Williams*.

[bb0175] Nussbaum M. (1997). Capabilities and human rights. *Fordham Law Review / Edited by Fordham Law Students*.

[bb0180] Nussbaum M. (2000). Women and human development: The capabilities approach.

[bb0185] Nussbaum M. (2002). Capabilities and disabilities: Justice for mentally disabled citizens. *Philosophical Topics*.

[bb0190] Nussbaum M. (2002). Capabilities and social justice. International Studies Review.

[bb0195] Nussbaum M. (2003). Capabilities as fundamental entitlements: Sen and social justice. *Feminist Economics*.

[bb0200] Nussbaum M. (2006). Frontiers of justice: Disability, nationality, species membership.

[bb0205] Nussbaum M. (2009). The capabilities of people with cognitive disabilities. *Metaphilosophy*.

[bb0210] Nussbaum M. (2011). Creating capabilities: The human development approach.

[bb0215] Nussbaum M., Sen, A. (Eds.). (1993). The quality of life. Oxford University Press..

[bb0220] Oliver M. (1983). Social work with disabled people.

[bb0225] Posner E.A. (2014). The twilight of human rights law.

[bb0230] Putnam D., Wasserman D., Blustein J., Asch A. (2013). Disability and justice (Stanford Encyclopedia of Philosophy).

[bb0235] Quinn G., Doyle S. (2012). Getting a life–living independently and being included in the community: A legal study of the current use and future potential of the EU structural funds to contribute to the achievement of article 19 of the United Nations convention on the rights of persons with disabilities.

[bb0240] Robeyns I. (2005). The capability approach: A theoretical survey. *Journal of Human Development*.

[bb0245] Robeyns I. (2011, April 14). The Capability Approach (Stanford Encyclopedia of Philosophy). https://plato.stanford.edu/entries/capability-approach/.

[bb0250] Robeyns I. (2017). Wellbeing, freedom and social justice: The capability approach re-examined.

[bb0255] Ruger J.P., Mitra S. (2015). Health, disability and the capability approach: An introduction. *Journal of Human Development and Capabilities*.

[bb0260] Scanlon T.M. (1998). What we owe to each other.

[bb0265] Sen A. (1979). Equality of what? Presented at the Tanner lecture on human values.

[bb0270] Sen A. (1983). Poor, relatively speaking. *Oxford Economic Papers*.

[bb0275] Sen A. (1992). Inequality reexamined. *Oxford University Press.*.

[bb0280] Sen A. (1994). Well-being, capability and public policy. *Giornale Degli Economisti e Annali Di Economia*.

[bb0285] Sen A. (1999). Choice, welfare and measurement.

[bb0290] Sen A. (1999). Commodities and Capabilities.

[bb0295] Sen A. (1999). Development as freedom.

[bb0300] Sen A. (2000). Social exclusion: concept, application, and scrutiny.

[bb0305] Sen A. (2002). Response to commentaries. Studies in Comparative International Development.

[bb0310] Sen A. (2009). The idea of justice.

[bb0315] Simplican S.C., Leader G., Kosciulek J., Leahy M. (2015). Defining social inclusion of people with intellectual and developmental disabilities: An ecological model of social networks and community participation. *Research in Developmental Disabilities*.

[bb0320] Stewart F. (2001). Women and human development: The capabilities approach, by MARTHA NUSSBAUM (Cambridge university press, Cambridge: 2000, pp. xxi +312, hbk £17.95). *Journal of International Development*.

[bb0325] Stewart F. (2005). Groups and capabilities. *Journal of Human Development*.

[bb0330] Szmukler G., Bach M., e20 (2015). Mental health disabilities and human rights protections.

[bb0335] Szmukler G., Daw R., Callard F. (2014). Mental health law and the UN convention on the rights of persons with disabilities. *International Journal of Law and Psychiatry*.

[bb0340] Tasioulas J. (2017, April 11). Are human rights anything more than legal conventions?. https://aeon.co/ideas/are-human-rights-anything-more-than-legal-conventions.

[bb0345] Trani J.-F., Dubois J.-L., Régis Mahieu F. (2011). Capability and disability: Approaches for a better understanding of disability issues. *Development efforts in Afghanistan: Is there a will and a way? The case of disability and vulnerability*.

[bb0350] UN. (2007). From exclusion to equality: Realizing the rights of persons with disabilities.

[bb0355] UN. (2018). The United Nations and disability: 70 years of the work towards a more inclusive world.

[bb0360] UNCRPD (2006). OHCHR | Convention on the Rights of Persons with Disabilities. http://www.ohchr.org/EN/HRBodies/CRPD/Pages/ConventionRightsPersonsWithDisabilities.aspx.

[bb0365] Uyan-Semerci P. (2007). A relational account of nussbaum’s list of capabilities. *Journal of Human Development*.

[bb0370] Vasbist L. (2010). Martha Nussbaum’s capability approach: Perils and promises. *Journal of the Indian Law Institute*.

[bb0375] Venkatapuram S. (2011). Health justice.

[bb0380] Venkatapuram S. (2013). Health, vital goals, and central human capabilities. Bioethics.

[bb0385] Venkatapuram S. (2014). Mental disability, human rights and the capabilities approach: Searching for the foundations. *International Review of Psychiatry*.

[bib412] Vizard P. (2010). The Idea of Justice*: Sen’s Treatment of Human Rights*. *Journal of Human Development and Capabilities*.

[bb0390] Vizard P., Fukuda-Parr S., Elson D. (2011). Introduction: The capability approach and human rights. *Journal of Human Development and Capabilities*.

[bb0395] Wasserman D., Asch A., Blustein J., Putnam D. (2012, July 6). Cognitive Disability and Moral Status (Stanford Encyclopedia of Philosophy). https://plato.stanford.edu/entries/cognitive-disability/.

[bb0400] Wells T. (2012). sen's capability approach. https://www.iep.utm.edu/sen-cap/.

[bb0405] Wilkinson D. (2008). Answering the challenge: Moral philosophy and the cognitively disabled. *Online Manuscript*.

[bb0410] Wolff J., de-Shalit, A. (2007). Disadvantage. Oxford University Press..

